# Increased duration of pollen and mold exposure are linked to climate change

**DOI:** 10.1038/s41598-021-92178-z

**Published:** 2021-06-17

**Authors:** Bibek Paudel, Theodore Chu, Meng Chen, Vanitha Sampath, Mary Prunicki, Kari C. Nadeau

**Affiliations:** 1 Sean N. Parker Center for Allergy and Asthma Research, Stanford University School of Medicine and Division of Pulmonary, Allergy, and Critical Care Medicine, Department of Medicine, Stanford University School of Medicine, Stanford, CA 94305 USA; 2 Department of Pediatrics, Stanford University School of Medicine, Stanford, CA 94305 USA

**Keywords:** Climate change, Environmental impact

## Abstract

Pollen and molds are environmental allergens that are affected by climate change. As pollen and molds exhibit geographical variations, we sought to understand the impact of climate change (temperature, carbon dioxide (CO_2_), precipitation, smoke exposure) on common pollen and molds in the San Francisco Bay Area, one of the largest urban areas in the United States. When using time-series regression models between 2002 and 2019, the annual average number of weeks with pollen concentrations higher than zero increased over time. For tree pollens, the average increase in this duration was 0.47 weeks and 0.51 weeks for mold spores. Associations between mold, pollen and meteorological data (e.g., precipitation, temperature, atmospheric CO_2_, and area covered by wildfire smoke) were analyzed using the autoregressive integrated moving average model. We found that peak concentrations of weed and tree pollens were positively associated with temperature (*p* < 0.05 at lag 0–1, 0–4, and 0–12 weeks) and precipitation (*p* < 0.05 at lag 0–4, 0–12, and 0–24 weeks) changes, respectively. We did not find clear associations between pollen concentrations and CO_2_ levels or wildfire smoke exposure. This study’s findings suggest that spore and pollen activities are related to changes in observed climate change variables.

## Introduction

Climate change, brought about by increased human activity in the last few decades, has a number of effects on planetary and human health^1^. Increased human activity has led to increases in a number of greenhouse gases such as carbon dioxide (CO_2_), methane (CH_4_), nitrous oxide (N_2_O), and ozone (O_3_). The global average atmospheric CO_2_ in 2018 was 407.4 parts ppm, which are higher than at any point in at least the past 800,000 years^2^. Global average temperature increased by about 1.0 °C from 1901 to 2016^3^ and continues to increase. The last 5 years, 2015–2019, have been the hottest years ever recorded. Climate change has led to increases in extreme weather events, such as increased flooding, wildfires, and thunderstorms^4^. The Centers for Disease Control and Prevention lists health effects of climate change including increased risk of atopic diseases such as allergic rhinitis and allergic asthma^5^. This trend is especially concerning due to the high prevalence of atopic disorders. Currently, approximately a quarter of individuals in developed countries^6^ are affected by allergic disease and these numbers are expected to increase with climate change. Temperature, rainfall, and other variables of climate change have been shown to indirectly effect allergies and asthma by their effects on pollen and molds^7,8^. Pollen from trees, grasses, and weeds and spores from mold are sources of allergens. Changes in vegetation, increased pollen/mold spore concentrations, and prolonged pollen seasons are linked to climate change. Increases in pollen/mold spores from climate change lead to allergies and asthma; the effects of climate change on human health is well documented^9^, especially in case of allergies^10^. For example, the distribution of the common ragweed (*Ambrosia artemisiifolia L*) has been expanding from Central to Northern and Eastern Europe due to changes in climate (rising temperatures, favorable precipitation) and that increases in CO_2_ has been increasing ragweed pollen production and allergies in these regions^11^. Following thunderstorms, a record-breaking number of visits to the emergency department for respiratory issues was observed in Australia in 2016^12^. During thunderstorms, whole pollen grains are swept into the clouds where they are broken up into smaller allergenic pollen fragments and eventually carried back to ground level^13^. These smaller size of pollen fragments permit their entry deep into the lungs. The mechanisms hypothesized for the fragmentation of pollen during thunderstorms include mechanical friction from wind gusts, electrical build up and discharge incurred during conditions of low relative humidity, and lightning strikes^14^. Air pollutants and CO_2_ levels have also been shown to affect the prevalence of aeroallergens^15^.

Airborne pollen and mold contribute significantly to adverse health outcomes in allergy and asthma. Increased pollen counts in spring is associated with increases in over-the-counter allergy medication sales and increases in emergency visits due to asthma exacerbations^16,17^. Pollen and molds are key triggers for allergic rhinoconjunctivitis and asthma flares. Increases in molds, caused by heavier rainfall and higher temperatures, can cause respiratory and asthma-related conditions as well as allergic bronchopulmonary aspergillosis, allergic fungal rhinosinusitis, and hypersensitivity pneumonitis^18^. There is also growing evidence that changes in the climate may be contributing to the rising incidence of food allergy due to changes in distribution of sensitizing plants and possibly due to a direct alteration in the allergenicity of plants with rising CO_2_ levels^19^. Since different patients are sensitive to different levels of atmospheric allergens, it is important to understand how the pollen and mold activities are changing over time. Patients who are sensitive to even small amounts of pollen and mold spores could benefit from the knowledge of their activities and outside peak allergy seasons, and how they vary with climate change.

As pollen and molds exhibit geographical variations, we sought to understand the effects of climate on common environmental pollens and mold spores in a specific region in the San Francisco Bay Area (Los Altos Hills, CA). In addition to measurements for maximum temperature, carbon dioxide level, and precipitation, we also compare the change in pollen or mold spore concentration with wildfire smoke exposure, as our area of study has been experiencing increasing exposure to wildfire smoke in recent years. An important gauge of the impact of climate change lies in phenology of pollen and mold exposure due to changes in pollen seasons and intensity of exposures^20–23^. We therefore evaluated a long-term dataset of outdoor pollen and mold observations over an 18-year period (2002–2019) using an in-depth analysis across the spectrum of aeroallergens (tree, grass and weed pollens and mold spores) contributing to allergic disease. The region is bounded by the Santa Cruz mountain range to the west and the San Francisco Bay to the east and has a Mediterranean climate. It is one of the most populated ecoregions in the United States and lies within the San Francisco-San Jose metropolitan area^24^. The average land change footprint of the area between 1973 and 2000 as determined by 11 land-cover classes (eg., mining, forest, agriculture, wetland) was estimated at 9.9%^24^. The vegetation is a mixture of grasslands, shrublands, and various forest types, the dominant among which is the evergreen forest^25^. In addition to the mixed evergreen forests, the coastal areas are covered by coastal scrub^26^. The region is undergoing changes in land cover due to rapid urbanization. A 19-year study (1984–2002) found that that the population increased by 30% and the urban area increased by 73%, leading to a 17% increase in impervious land cover and a 27% decrease in pervious surfaces^27^.

## Results

### Annual and seasonal trend analysis

A summary of the terminologies to measures pollen and spore activity are presented in Table 1 and described in Methods.

**Table 1:** Measures of pollen and spore activity, and their description.

Measure	Description	Unit
Pollen or Spore Concentration	Number of pollen or spore grains per unit volume of air collected over a period of 24 h	Pollen grains/m^3^
Weekly Average Concentration (WAC)	Pollen or Spore Concentration averaged by calendar week	Pollen grains/m^3^
Annual Average Concentration (AAC)	Pollen or Spore Concentration averaged by calendar year	Pollen grains/m^3^
Start of Season		Week of the year (1–52)
End of Season		Week of the year (1–52)
Season Length	Number of weeks between the end of season and the start of the season	Number of Weeks
Maximum Pollen or Spore Concentration (MPC)	Maximum value of WPCs over the whole year	Pollen grains/m^3^
Peak Week	Week when the WPC reaches its maximum value in a year	Week of the year (1–52)
Number of Active Weeks (NAW)	Number of weeks where concentration is greater than zero	Number of Weeks
Seasonal Pollen or Spore Integral (SPIn)	Sum of WACs over the season length	Week * Pollen grains/m^3^
Annual Pollen or Spore Integral (APIn)	Sum of WACs over the whole year	Week * Pollen grains/m^3^

For all three groups major allergens, selected species, and commonly observed species (Methods), we analyzed annual trends in pollen and mold concentrations, seasonality, and activity. All statistical analyses were performed in the Python programming environment (Python Software Foundation, http://www.python.org ) and *p* values < 0.05 were considered statistically significant.

Summary statistics for annual and seasonal characteristics of major allergens is presented in Table 2. The week on which pollen concentrations peak for each type of allergen is given in the “Peak Week” column, which shows the distinct seasonal pattern of each type of major allergen. Tree pollens peak in Spring, Grass pollens peak in late Spring and early Summer, Weed pollens peak in Summer, and Mold concentrations peak in Fall. To quantify various annual trends for each observation, the annual average values for major allergens as well as the annual trends for the different climate variables were analyzed and plotted (Supplementary Figs. S1 and S2). Statistical significance was calculated by fitting linear trends using first-order linear regression. Supplementary Fig. S3 shows statistically significant increasing trends for T_Max_ and CO_2,_ while there was no such trend for precipitation. In Supplementary Table S1-S3, we present the temporal trends for major allergens, selected species, and most commonly observed species. A decreasing trend for major allergens’ annual average concentrations (statistically significant for trees and grasses, coefficients of linear trend: − 3.16 and − 0.19 respectively) were observed (Supplementary Table S1). Although the annual average concentrations for all major allergens except weeds showed a decreasing trend, only tree and grass pollens were statistically significant with *p* value < 0.05. For details about individual tree, weed, grass, and mold species refer to Fig. 2 and Supplementary Tables S3-S5. We also analyzed changing season length for different pollen and mold spore types over the years. We found increases in the season length for tree pollens (0.38 weeks). Given the increasing season lengths for some pollen despite the decrease in average annual pollen counts, the number of active weeks was also investigated. The annual linear trends for these values are shown in the third column of Supplementary Table S1-S3. The number of active weeks significantly increased for tree pollens and molds. To examine whether pollen and mold seasons were starting sooner and extending further into the year, the weeks of the year when the pollen seasons and mold seasons start and end were calculated (seasons were calculated using an established procedure, Methods); the coefficients of linear trends are shown in the fourth and fifth columns of Supplementary Tables S1-S3. For tree pollens, we observed a significant delay in the end of season (0.29 weeks).

**Table 2:** Summary statistics for major allergens (2002–2019), showing information about seasonality, number of active weeks (NAW), Seasonal Pollen Integrals (SPIn), Annual Pollen Integrals (APIn), Maximum Pollen Concentrations (MPC), and Peak Week. Mean, Standard Deviation (SD), Minimum, and Maximum values are shown for each measure.

Type		Season length (Number of weeks)	Start of season (Week of the Year)	End of season (Week of the year)	NAW (Number of weeks)	SPIn	APIn	MPC	Peak week
**Trees**	Mean	40.39	18.0	46.33	38.89	4434.89	5060.25	1,035.11	12.78
	SD	3.70	5.94	3.24	4.10	1234.02	1324.63	486.83	2.6
	Min	34.0	3.0	40.0	32.0	2667.45	2811.48	327.87	7.0
	Max	47.0	10.0	52.0	46.0	7628.81	8403.98	2215.46	18.0
**Weeds**	Mean	20.94	19.39	40.33	17.61	73.58	83.63	15.55	27.94
	SD	5.02	2.68	4.24	3.45	33.53	32.02	10.39	7.53
	Min	14.0	13.0	33.0	11.0	28.10	39.81	4.68	18.0
	Max	31.0	25.0	50.0	23.0	177.99	185.01	49.18	48.0
**Molds**	Mean	45.83	3.17	49.0	40.17	113,907.89	123,278.93	22,539.71	33.39
	SD	4.48	1.89	3.50	4.59	51,913.13	53,000.61	19,930.37	18.03
	Min	36.0	1.0	40.0	32.0	49,590.08	53,597.63	5409.92	3.0
	Max	50.0	7.0	52.0	47.0	210,622.98	216,993.50	89,644.01	51.0
**Grasses**	Mean	14.56	14.56	29.11	15.11	162.45	172.83	41.37	19.56
	SD	5.96	2.75	4.40	3.23	89.85	88.39	30.88	2.25
	Min	7.0	7.0	25.0	10.0	62.06	64.40	11.71	15.0
	Max	27.0	18.0	40.0	20.0	371.0	374.12	131.15	24.0

**Figure 1:**
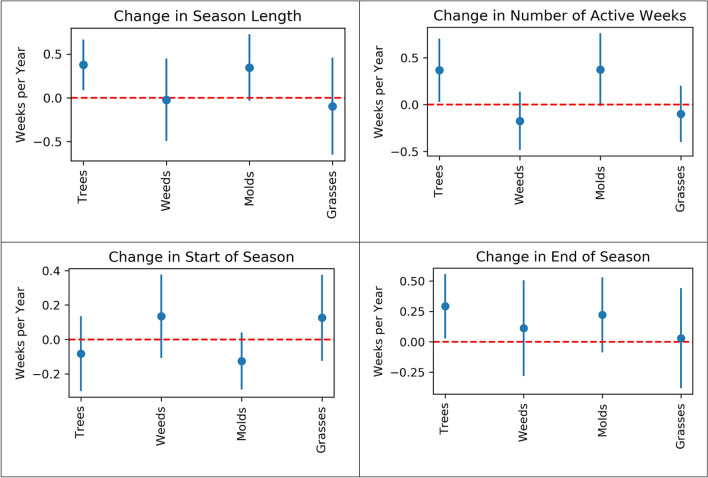
Coefficient estimates and 95% confidence intervals for change in season length, number of active weeks, start of season, and end of season for Major allergens.

**Figure 2:**
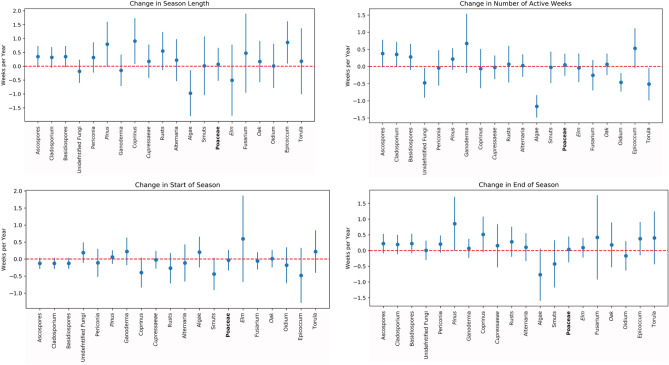
Coefficient estimates and 95% confidence intervals for change in season length, number of active weeks, start of season, and end of season for the most commonly observed species. Italics: *Trees*, Normal: Molds, Bold: **Weeds.**

Supplementary Tables S2 and S3 reveal interesting properties regarding annual concentrations, which are different from what was observed for major pollens and mold. For both mold and weed species, there were increases in annual average concentrations (although not statistically significant), while all tree species show a decreasing trend. The pollen season is getting longer and starting earlier for a majority of species, but the trends were not statistically significant. Similarly, the season is ending later for a majority of species, but the trend was not statistically significant. In Supplementary Table S3, the most commonly observed species (all of which are molds) demonstrate increasing trends for the number of active weeks. The top-two most commonly observed species were active for an average of half a week more than prior years. In Figs. 1 and 2, we visually show the change in seasonal characteristics and number of active weeks for major allergens, and commonly observed species. In Supplementary Fig. S1, we visually show these results for those selected species whose season length we were able to calculate for at least 10 years during our study duration.

### Association of pollen counts with climate variables

To study the association of pollen concentrations with patterns of climate variables, the well-established autoregressive method Auto Regressive Integrated Moving Average (ARIMA) was used. For details on ARIMA, please refer to the Methods section.

Associations of pollen and mold with three climate variables (maximum temperature, precipitation, carbon-dioxide, and smoke area) are shown in Table 3. The columns in the table show the association of climate variables in different lags, e.g., T_Max_ (0–24) shows the association of maximum temperature in prior six months on the pollen and mold concentrations. In other words, these values show how peak pollen and mold concentrations are related to the lagged values of different climate variables. Climate variables immediately before, as well as a year before could be strongly associated with pollen and mold concentrations as shown in the results.

**Table 3:** Summary of the multivariate ARIMA model with the explanatory climate variables for major species (2002–2019). The best fitting univariate ARIMA( , , )( , , , ) model parameters were used to estimate the coefficients and p-values for different lag of the climate variables: Smoke Area, CO_2_, precipitation, and T_MAX_. AIC denotes the Akaike’s Information Criterion. Lags indicate averaged values at prior indicated durations, divided into week 0–1, week 0–4, week 0–12, week 0–24, week 0–52, and week 53–104 for immediate, short-term, seasonal, and pre-seasonal effects. Numbers in bold represent the statistically significant associations(*p* value < 0.05).

Species	Variable	Lag (weeks)	Coefficient	*p* value	AIC
Trees	**T**_**MAX**_	**0–1**	**4.62**	**0.0**	**10,006.94**
Trees	T_MAX_	0–4	1.37	0.64	10,022.7
Trees	**T**_**MAX**_	**0–12**	**− 11.42**	**0.0**	**9983.15**
Trees	**T**_**MAX**_	**0–24**	**− 15.61**	**0.0**	**9990.79**
Trees	T_MAX_	0–52	1.52	0.9	10,020.66
Trees	T_MAX_	53–104	1.54	0.83	10,020.57
Trees	**Precipitation**	**0**	**− 325.34**	**0.0**	**10,021.76**
Trees	**Precipitation**	**0–4**	**1647.64**	**0.0**	**10,058.66**
Trees	**Precipitation**	**0–12**	**2671.15**	**0.0**	**9996.46**
Trees	**Precipitation**	**0–24**	**3033.11**	**0.0**	**9997.49**
Trees	Precipitation	0–52	3086.19	0.05	10,015.39
Trees	Precipitation	53–104	3062.31	0.18	10,018.61
Trees	CO_2_	0	0.28	0.86	10,019.79
Trees	CO_2_	0–4	0.28	0.86	10,019.88
Trees	CO_2_	0–12	0.28	0.86	10,019.94
Trees	CO_2_	0–24	0.28	0.86	10,020.23
Trees	CO_2_	0–52	0.28	0.87	10,020.06
Trees	CO_2_	53–104	0.28	0.88	10,020.1
Trees	Smoke Area	0	− 0.0	0.9	5331.31
Trees	Smoke Area	0–4	− 0.01	0.64	5330.46
Trees	Smoke Area	0–12	− 0.06	0.3	5326.92
Trees	Smoke Area	0–24	− 0.07	0.43	5328.85
Trees	Smoke Area	0–52	− 0.05	0.58	5330.38
Trees	Smoke Area	53–104	− 0.1	0.56	5329.56
Weeds	**T**_**MAX**_	**0–1**	**0.12**	**0.0**	**3986.07**
Weeds	**T**_**MAX**_	**0–4**	**0.15**	**0.0**	**3984.21**
Weeds	**T**_**MAX**_	**0–12**	**0.13**	**0.02**	**3999.08**
Weeds	T_MAX_	0–24	− 0.07	0.33	4012.34
Weeds	**T**_**MAX**_	**0–52**	**0.6**	**0.03**	**4002.36**
Weeds	T_MAX_	53–104	− 0.15	0.43	4012.91
Weeds	Precipitation	0	− 3.74	0.59	4009.71
Weeds	Precipitation	0–4	− 12.59	0.15	4004.0
Weeds	Precipitation	0–12	− 21.29	0.06	4003.29
Weeds	Precipitation	0–24	13.25	0.29	4012.36
Weeds	Precipitation	0–52	− 39.32	0.32	4011.78
Weeds	Precipitation	53–104	53.8	0.21	4010.65
Weeds	CO_2_	0	0.0	0.88	4013.84
Weeds	CO_2_	0–4	0.0	0.88	4013.65
Weeds	CO_2_	0–12	0.01	0.87	4013.41
Weeds	CO_2_	0–24	0.01	0.83	4013.41
Weeds	CO_2_	0–52	0.0	0.88	4013.81
Weeds	CO_2_	53–104	0.01	0.71	4013.65
Weeds	Smoke Area	0	0.0	0.07	2203.32
Weeds	Smoke Area	0–4	0.0	0.24	2198.29
Weeds	Smoke Area	0–12	− 0.0	0.82	2199.8
Weeds	Smoke Area	0–24	− 0.0	0.35	2198.58
Weeds	Smoke Area	0–52	0.0	0.18	2197.21
Weeds	Smoke Area	53–104	0.0	0.88	2199.68
Molds	T_MAX_	0–1	32.86	0.53	15,099.83
Molds	T_MAX_	0–4	34.27	0.59	15,097.05
Molds	T_MAX_	0–12	37.8	0.48	15,091.41
Molds	T_MAX_	0–24	39.14	0.61	15,085.65
Molds	T_MAX_	0–52	38.46	0.89	15,089.46
Molds	T_MAX_	53–104	39.06	0.85	15,087.79
Molds	**Precipitation**	**0**	**33,152.72**	**0.0**	**15,082.19**
Molds	**Precipitation**	**0–4**	**53,504.09**	**0.0**	**15,032.64**
Molds	**Precipitation**	**0–12**	**51,294.37**	**0.0**	**15,085.86**
Molds	**Precipitation**	**0–24**	**51,265.07**	**0.0**	**15,106.74**
Molds	Precipitation	0–52	74,853.95	0.12	15,081.06
Molds	Precipitation	53–104	69,574.0	0.17	15,102.74
Molds	CO_2_	0	6.93	0.81	15,089.69
Molds	CO_2_	0–4	6.88	0.82	15,089.75
Molds	CO_2_	0–12	6.87	0.82	15,089.93
Molds	CO_2_	0–24	6.89	0.82	15,089.74
Molds	CO_2_	0–52	6.95	0.82	15,089.55
Molds	CO_2_	53–104	7.02	0.83	15,089.46
Molds	Smoke Area	0	− 0.29	0.89	8079.46
Molds	Smoke Area	0–4	− 0.05	0.96	8080.52
Molds	Smoke Area	0–12	0.78	0.67	8081.13
Molds	Smoke Area	0–24	1.98	0.13	8081.2
Molds	Smoke Area	0–52	1.84	0.49	8084.52
Molds	Smoke Area	53–104	3.34	0.4	8083.22
Grasses	**T**_**MAX**_	**0–1**	**0.14**	**0.04**	**5014.98**
Grasses	T_MAX_	0–4	0.09	0.62	5024.0
Grasses	T_MAX_	0–12	− 0.16	0.49	5022.6
Grasses	**T**_**MAX**_	**0–24**	**− 0.87**	**0.0**	**4980.78**
Grasses	T_MAX_	0–52	− 0.43	0.59	5024.5
Grasses	**T**_**MAX**_	**53–104**	**− 1.22**	**0.0**	**5013.59**
Grasses	Precipitation	0–1	− 4.29	0.67	5023.38
Grasses	Precipitation	0–4	− 14.7	0.46	5022.87
Grasses	Precipitation	0–12	20.92	0.45	5023.92
Grasses	**Precipitation**	**0–24**	**165.51**	**0.0**	**4984.91**
Grasses	Precipitation	0–52	99.38	0.37	5023.58
Grasses	Precipitation	53–104	173.24	0.05	5021.09
Grasses	CO_2_	0–1	0.01	0.88	5025.06
Grasses	CO_2_	0–4	0.01	0.89	5025.08
Grasses	CO_2_	0–12	0.01	0.89	5025.2
Grasses	CO_2_	0–24	0.01	0.89	5025.35
Grasses	CO_2_	0–52	0.01	0.89	5025.41
Grasses	CO_2_	53–104	0.01	0.9	5025.44
Grasses	Smoke Area	0–1	− 0.0	0.69	2746.74
Grasses	Smoke Area	0–4	− 0.0	0.76	2746.64
Grasses	Smoke Area	0–12	− 0.0	0.61	2743.71
Grasses	Smoke Area	0–24	− 0.01	0.46	2739.41
Grasses	Smoke Area	0–52	0.0	0.98	2748.4
Grasses	Smoke Area	53–104	− 0.0	0.76	2748.19

The strongest association with recent temperature changes was observed in the concentrations of tree and weed pollens. For tree pollens, the association is positive with changes in temperatures in the same week (lag 0–1), whereas the association is negative with changes in temperatures in longer timeframes (lag 0–12 and 0–24). For weed pollens, the associations are positive for both immediate, seasonal and annual timeframes (lag 0–1, 0–4, 0–12, 0–52). Results in Supplementary Table S1 revealed that tree and weed pollens peak in Spring and Summer respectively. This suggests that for trees, their peak pollen concentrations are associated with rising Spring temperatures (likely associated with blooming season) and falling Winter temperatures. Similarly, for weeds, their peak pollen concentrations are associated with rising summer and spring temperatures.

Peak values of tree pollen concentrations were also associated with lagged values of precipitation (negative at lag 0–1 and positive at lag 0–4, 0–12, 0–24). This suggests that Winter rains are associated with increased tree pollen concentrations a few weeks later in Spring, but decreased tree pollen concentrations week immediately after. For details about individual tree, weed, grass, and mold species refer to Fig. 2 and Supplementary Tables S3-S5.

Mold concentrations were also observed to be significantly associated with lagged values of precipitations (lag 0–1, 0–4, 0–12, 0–24), suggesting that increased rainfall leads to increase in mold spores up to six months in the future. In the case of grasses, whose pollen concentrations peak in late Spring and early Summer, the association was found to be strongest with lagged values of temperature and precipitation from up to 6 months in the past (lag 0–24). This suggests that increase in Winter rain or decrease in Winter temperature are associated with higher grass pollen concentrations in the next season. Grass pollens were also observed to be negatively associated with lagged values of temperature from the previous year (lag 53–104) and positively associated with temperature of the same week. This suggests that increase in Summer temperature are associated with higher grass pollens in the same week.

In this dataset, strong associations between atmospheric CO_2_ and pollen and mold counts were not observed. Previous studies also found it difficult to separate the influence of rising CO_2_ from temperature change on growth or floral phenology of plants^28^. In our study, wildfire smoke exposure was also not found to be associated with pollen or mold concentrations for any of the major allergens.

## Discussion

In this retrospective analysis of pollen and mold concentrations in the San Francisco Bay Area during the past two decades, we observed that whereas average concentrations for most species is decreasing over time, the season length and number of active weeks are increasing. Further, these observations are statistically significant for the most commonly observed species and are also correlated with observed maximum temperature and precipitation in the region. While previous studies in this subject have looked at a limited number of species, our analysis covers more than twenty species observed in the studied region for a long time-period.

Some of our findings are consistent with the observations made in other studies. These include increasing pollen seasons, and their association with observed climate variables. However, we found that the average annual concentrations of most species in our study region has been decreasing over the years. Prior studies with regards to annual trends of pollen concentrations show a mixed result, with increases in some areas and decreases in other. Notably, a study of pollen counts in different areas in the United States^29^ observed that the annual concentrations were increasing significantly in northern latitudes, but not in the southern latitudes. In our study, we observed increasing periods of activity for several species even as we observed a decrease in their average annual concentrations, suggesting that the pollen and mold activities are increasing outside their peak seasons. Rapid urbanization and land-use change could be a possible reason for decreasing trend of pollen concentration in the area under our study. Moreover, changes in climate variables like temperature could be due to both local change in land-use (e.g., urbanization) or global climate pattern. Both climate change and land-use change could bring about changes in the species of trees and plants in a region due to species migration or changes in architecture and landscaping preferences^28^.

While indoor molds are known to be present throughout the year, our study concerns outdoor molds, whose season peaks in Summer and Fall^30^ and are known to cause allergic reactions. In the region of our study, we observed that mold species are the most commonly observed ones, and both the season length and number of active weeks for the most frequent among them have increased in the past two decades.

A major difference of our study in comparison to previous studies is the wide range of pollen and mold species covered in our analysis. Additionally, we also look at the changing trends for the number of active weeks of pollen and mold, in addition to their seasons. Seasons are the durations when pollen and mold concentrations reach their peaks. However, pollens and molds are active outside of those peak durations as well and knowing how these activities are changing could be beneficial to improve care for specific groups of people. Antihistamine and anti-inflammatory allergy medications can take up to 4 weeks to be fully effective^31^. Because individuals could be sensitivity to even small amounts of pollens and molds, our study could help both patients and physicians prepare ahead of peak seasons.

The relationship between climate change and phenology in a variety of plant species has been an area of increasing interest^22,32^. Previous studies have shown an advancement in the onset of pollen seasons in plants^33,34^. The U.S. Environmental Protection Agency has acknowledged the role of changing climate on pollen season^35^. A recent study using more than 20 years of airborne pollen data from across 13 countries in the Northern hemisphere demonstrated the effect of changing temperature on pollen season and load^28^. The International Phenological Gardens, a European network, has reported that since the 1960s, growing seasons have increased by approximately 11 days^20^. Ziello et al. reported an increase in atmospheric pollen of multiple types between 1977 and 2009 across Europe^36^.

Previous studies have shown that temperature and water availability correlate with pollen pro-duction intensity^37,38^. Increases in temperature directly increases pollen production both in the year prior to the pollen seasons, as well as in the month preceding flowering. A study from Spain examining the pollen trends of olive trees found increases in temperature were correlated with an earlier start and a later end to the pollen season each year between 1982 and 2011, demonstrating an increase in pollen production. Modeling suggested significant changes in the reproductive cycle of the olive tree due to climate change^33^. Several studies have demonstrated a relationship between higher temperatures and sun exposure the year prior to higher daily pollen concentrations the following year^39,40^. The previous summer’s temperature influences the intensity of pollen production as pollen grains are being formed the year prior, which depend on the photosynthates from the summer to reproduce in the spring. Studies have also found that higher temperatures in the month leading up to flowering also directly correlated with higher pollen concentrations^41,42^. Fungal spore concentrates increase with increased temperature^43^.

The relationship between rainfall, water availability and the concentration of pollen has been variable. Soil moisture is needed for seed germination but precipitation during flowering and pollen dispersal can wash out pollen and lower counts. Water deficits have been shown to delay olive flowering^44,45^. Drought conditions have been shown to decrease pollen in Switzerland and the Mediterranean^46,47^. In North America, tree pollen increases with increasing precipitation. However, Rasmusseen found that precipitation from the previous year was negatively correlated with average birch pollen concentration; although this was postulated to be due to a negative correlation between temperature and precipitation^45^. Increased water and soil moisture stimulate fungal growth spore growth and dissemination.

In our study, we also found significant associations of temperature and precipitation with pollen activities of multiple pollen species, consistent with prior work in this area. Additionally, this work sheds light into the role of changing climate with regard to individual species, as well as their short- and long-term influences (a few weeks to a year). Since different geographical areas have different prevalence of plant species that contribute to pollen activity, this analysis helps understand the unique characteristics of the San Francisco Bay Area’s pollen seasons and their changing nature.

CO_2_ is the source of carbon for photosynthesis. Ziello et al. suggested that that rising CO_2_ concentrations may be responsible for pollen increases^36^. Increases in CO_2_ is also thought to contribute to mold growth. Zhang et al. used Bayesian modeling and found that annual mean CO_2_ concentrations were significantly related to birch pollen levels and projected rising pollen counts in the next century^29^. Growth chamber experiments in which trees, grasses and weeds are exposed to higher levels of CO_2_ show increase in pollen production^48–50^. Experiments have also shown that increasing CO_2_ increases mold spore production^51^. However, it has also been noted that ascertaining the influence of rising carbon dioxide apart from temperature on pollen activity is hard to ascertain^28^. In our study, with regards to climate variables, as expected, we found that both CO_2_ and maximum temperature shows statistically significant increasing trends. For the pollen and mold types we studied in the San Francisco Bay Area, we found that the annual average concentrations show a decreasing trend over the years with grass pollens and some frequently occurring molds and tree pollens showing statistically significant trends. For trees and several molds, the average number of active weeks shows a strong increasing trend over the years.

Future changes due to climate change are expected to further impact pollen production. Hamaoui-Laguel et al., using models to predict ragweed pollen concentrations in Europe found an anticipated four-fold increase in airborne pollen levels by 2050^52^, which has been predicted to increase rates of pollen sensitization^53^. Similar results have been found in Italy with increasing tree pollen counts and an associated increase in patients sensitized to pollen^54^. Better understanding the impact of climate change on pollen and mold spore production can guide predictive modeling to forecast pollen and mold production, improving public health measures to prevent asthma and allergy flares and prepare resources to respond to events that cause spikes in pollen and mold levels.

A limitation of this study was that we used a single site of pollen and mold collection and analysis. As pollen and mold spore concentrations are influenced by changes in the local environment and changes in landscaping, additional sites of collection would further strengthen the reliability of the data and interpretations. Thus, the results of this study provide insight into only the local region of the San Francisco Bay Area. The decreasing annual average concentrations for pollens and molds could be due to several of these reasons, including the rapid urbanization and change in vegetation cover in the area of our study. However, the findings are consistent with other studies examining phenology and climate change and suggest broad implications and a global impact of climate change on allergen activity.

Given the observational nature of the study, multiple environmental factors may be contributing to the observed findings. Given the complicated nature of plant biology, other factors are difficult to account for such as masting behavior, and the production of many seeds by a plant. Furthermore, local atmospheric changes and soil composition on pollen activity may have influenced our findings, but these variables could not be tested due to the lack of a suitable dataset. In addition to smoke exposure, particulate matter could also have influenced pollen activity and should be evaluated in future studies.

In future studies, we plan to examine the change in pollen concentrations and activities and their relationships with clinical outcomes. By combining datasets of electronic health records (EHRs), we could study how changing climate patterns and pollen activities affect patient visits as well as prescription of allergy medications. Additionally, datasets of land cover could be used to study the association between change in land-use with the changes in the activities and concentrations of pollens and molds. Although the most commonly observed species are specific to the study location, future studies could look into how the activities of some of the species observed in our study area have changed in other similar geographical locations across the world.

Extant research has largely focused on individual species or on a few taxa. We provide detailed analysis of pollen and mold activity for the twenty most frequent species in the area of our study, as well as for selected species of clinical significance. The long temporal span of this dataset (18 years) lends itself to studying the effect of changing climate on pollen and mold activity. As temperatures are increasing, the length of the pollen season for several species is significantly increasing. Similarly, there are strong associations between multiple pollen and mold species and climate variables, although for some species the direction of these association is not always uniform.

## Methods

### Collection and counts of pollen and mold spores

We used a database spanning 18 years (2002–2019) of weekly pollen and mold spore concentrations for an area in the San Francisco Bay Area (Los Altos Hills, Santa Clara County, CA, USA) obtained from a National Allergy Bureau (NAB) certified pollen counting station. The location of the pollen collection site and neighboring areas in the San Francisco Bay Area is shown in Supplementary Fig. S5. Concentrations of outdoor pollens and mold spores were obtained with a Burkard Spore Tap (Burkard Collector) and were identified by species and also categorized as tree pollen, grass pollen, weed pollen or mold spore. The Burkard Collector is a volumetric air sampler and a standard device for monitoring airborne pollen and spores. This device draws in air at regular intervals and as a result, any airborne particles with enough inertia are captured on a surface inside the device, e.g., a greased tape or a microscopic slide. The capturing surface moves in a steady speed, allowing for newer samples to be collected. The device also has a wind vane and an ability to rotate, making it always oriented into the wind. The Burkard Collector can collect particles up to 3.7 µm and has been used in prior studies^55^.

Time Series Analysis (ARIMA).

ARIMA is a well-established method for time-series analysis and has been used to find associations between climate variables and health outcomes^56^. The pollen timeseries datasets have a seasonal component, as can be observed in the decomposed time series plots (Supplementary Figs. S6-S9). For more details on time-series decomposition, see Supplementary Appendix Section “Time Series Decomposition.” For this reason, we used SARIMA, which is the seasonal variation of ARIMA, and which has the flexibility to control the seasonality and autocorrelation in the timeseries. In ARIMA( , , ) models, the target variable is predicted using three components: (1) past values (lags) of the target variable (AR or autoregressive), (2) differentiation of the timeseries, and (3) a moving average model (MA or moving average) on past forecast errors. The parameters for these three components together define the order of an ARIMA(p, d, q) model, where p, d, q correspond to the first, second, and third components, respectively. The seasonal ARIMA(p, d, q) (P, D, Q, m) model has an additional seasonal order where the parameters P, D, Q similarly refer to the seasonal variants of the first, second, and third components, and m refers to the frequency of the timeseries. This model is written in short as ARIMA(, , ) ( , , , ). All statistical analyses were performed in the Python programming environment (Python Software Foundation, http://www.python.org ) and *p* values < 0.05 were considered statistically significant. In all ARIMA models, the Box-Ljung test was used to test the null hypothesis that the autocorrelations of the residuals equal zero and the augmented Dickey–Fuller test was used test whether the timeseries was stationary.

First, univariate ARIMA( , , )( , , , ) models of different orders were fitted for the timeseries of pollen and spore concentrations of each major allergen (Trees, Weeds, Molds, Grasses) using the Box-Jenkins approach^57^. Additional information on species can be obtained from Tables S3/S4. The best performing ARIMA models for each allergen were chosen based on the Akaike Information Criterion (AIC), and they are presented in Table 4.

**Table 4:** Summary of the univariate ARIMA( , , )( , , , ) model fitting parameters on the timeseries datasets for major allergens (2002–2019). The best fitting models are chosen based on Akaike’s Information Criterion (AIC).

Species	Order	Seasonal Order	AIC
Trees	(3, 0, 0)	(0, 0, 1, 52)	10,761.74
Weeds	(1, 0, 1)	(0, 0, 1, 52)	4271.35
Molds	(1, 0, 0)	(0, 0, 0, 52)	16,119.25
Grasses	(1, 0, 1)	(0, 0, 0, 52)	5673.3

Next, the best fitted ARIMA model was examined together with different climate variables. The statistical significance of the climate variables was then determined using these multivariate ARIMA models. Given prior finding in the literature than pollen activity can be influenced by climate factors from earlier seasons, climate variables at different lags (earlier periods) were included to check the associations of immediate, short-term, seasonal, and pre-seasonal climate variations with peak pollen and spore concentrations. The values of each climate variable were averaged for the following lagged durations: week 0–1 (immediate), week 0–4 (short-term), week 0–12 (seasonal), week 0–24 (pre-seasonal), week 0–52 (annual), week 53–104 (previous year).

### Environmental data

Environmental data were collected from a variety of databases. These environmental variables and datasets have been used in prior studies on the environmental health^58,59^. The daily maximum temperature T_MAX_ (measured in Fahrenheit) and precipitation data (measured in inch) were collected from the National Climatic Data Center of the National Oceanic and Atmospheric Administration (NCDC/NOAA). NCDC publishes historical climate observations for several monitoring sites across the United States. The San Jose monitoring site was selected because of its proximity to the site of the pollen and mold spore collection and as it had coverage spanning the period (2002–2019) during which the pollen and mold spore data were collected. For atmospheric CO_2_ data, none of the monitoring sites in California had observations for the complete period (2002–2019); therefore, the CO_2_ dataset (measured in parts per million) from Mauna Loa Observatory (MLO) of NOAA, located in Kona, Hawaii was used. This dataset informs us about the changes in CO_2_ trends in the earth’s atmosphere. As a cross-check, we compared the correlation of the MLO dataset with the CO_2_ observations during 2008–2017 from the Humboldt State University observatory in Northern California. We found a correlation coefficient of 0.85 for the 7-day moving averages in the two datasets. These datasets are overlaid in Supplementary Fig. S10 and the linear relationship between these two datasets is shown in Supplementary Fig. S11, revealing a highly linear trend. For data on wildfire smoke exposure, we utilize the Hazard Mapping System (HMS) dataset developed by the National Oceanic and Atmospheric Administration (NOAA) of the United States government. On a daily basis, trained analysts use visible satellite imagery, satellite-based automatic fire detections, and infrared images to annotate fire locations and perimeter of smoke plumes^60^. Additionally, they also annotate the amount of smoke density (as low, medium, or high) and this dataset is available from 2010 onwards. From the daily dataset of smoke plume perimeters, we first identified smoke plumes that intersected with Santa Clara county. For those intersecting smoke plumes, we calculated the total area of smoke plume that lied wholly inside the county boundaries, resulting in a daily time series containing the area of smoke plumes that the county was exposed to.

### Dataset preparation

The pollen and mold observations include weekly concentration of several commonly observed species in the collection area, although some weeks contain more than one observation. Those weeks with no pollen counts were treated as missing data. The pollen and mold observations were resampled to obtain a dataset with weekly concentrations. In addition, three datasets of pollen and mold spore concentrations were extracted from the raw observation files. The first dataset, called “Major allergens”, summarizes the concentrations for four major pollen and mold categories: trees, grasses, weeds, and molds. The second dataset (“Most-active species”) includes the concentrations for the twenty most active species. To identify the most-active species, the species were ordered by the number of total weeks in which each species had a concentration greater than zero during the complete observation period (2002–2019). Then, the top twenty species were selected from this list. The third dataset (“Selected species”) includes seven species which were picked based on their known importance in allergic outcomes^61,62^. Unlike pollen and mold concentrations which have a weekly frequency, the climate variables have a daily frequency. Moving seven-day averages of the climate observations were created to help offset the effect of short-term measurement effects and outliers, which is a standard practice in time-series analysis^63^. For smoke plumes we converted the daily timeseries to a weekly one by taking 7-day maximum area of smoke exposure. Finally, the weekly timeseries from the climate datasets were overlaid with the three pollen and mold spore datasets based on their dates. This generated combined time-series datasets of pollen and mold concentrations with corresponding climate variables. The annual average values for major allergens and different climate variables are plotted in Supplementary Figs. S1 and S2.

### Pollen species

We found over 100 different species of pollen and mold spores in our dataset, which are listed in Supplementary Table S5. Since some species are observed more often than others, a list of 20 most commonly observed species was created. To create this list, each species was ordered by the number of weeks in which it had a concentration greater than zero. The list of 20 most commonly observed species is shown in Supplementary Table S4. Similarly, owing to the clinical importance of some, a list of “selected species” was created containing *Alternaria* spp., *Penicillium/Aspergillus*, *Quercus* spp. (Oak), *Cupressaeae* spp. (incl. junipers/cedars), *Betulaceae* spp. (birch), *Artemesia* spp. (sage), and *Ambrosia* spp.*/Franseria* spp. (ragweed). Additionally, the counts for the four major pollen and mold were summarized as: trees, grasses, weeds, and molds.

### Concentrations and activity for pollens and mold spores

Pollen or mold spore *concentrations* refer to the observations by the counting device during a given time period. When the concentrations are annually averaged, we call them *annual average concentrations* (AAC) and when they are weekly averaged, we call them *weekly average concentrations* (WAC). Seasonal and Annual Pollen or Spore *integrals* refer to sums of WAC values over a season (SPIn) and calendar year (APIn), respectively.

Further, we differentiate between weeks with pollen and mold spore concentrations greater than zero with those when the pollen and spore concentrations are zero. We refer to the weeks where the pollen and spore concentrations are greater than zero as *active* weeks and the total number of active weeks in a calendar year as Number of Active Weeks (NAW). In other words, *active weeks* correspond to duration, whereas *integrals* correspond to the quantitative extent of that activity. Finally, a pollen or mold *season* is the continuous period during a calendar year when their observations are most concentrated. Each season has a starting week and an ending week, and the *season length* refers to the encompassing number of weeks. To calculate season length, we followed the procedure described in Ziska et al.^28^. To identify the start of the season, we take the first continuous 4-week period of the year when the concentrations are greater than zero and take the last week of that 4-week period. To identify the end of the season, we take the last continuous 4-week period of the year when the concentrations are greater than zero and take the last week of that 4-week period. For some species on some years, there were no continuous 4-week period with concentrations greater than zero. In those cases, we took a second approach and considered the fourth week when the concentrations are greater than zero as the start of the season and fourth-from-the-end week when the concentrations are greater than zero as the end of the season. Even after this procedure, for some species on some years, we observed less than 4 weeks when the concentrations are greater than zero. For those, we took a third approach and considered the first week when the concentrations are greater than zero as the start of the season and the last week when the concentrations are greater than zero as the end of the season. In Figs. 1–2 and Supplementary Fig. S4, only those species for which we could calculate the season using first or second approach for at least 10 years are shown.

Consider the following example to illustrate the differences between these measures. If in a given year pollen concentrations are greater than zero on weeks 8, 15, 16, 17, 18, 20, 22, 25, 26, 27, 28, 35, the value of NAW is 12. The value of APIn is the sum of pollen concentration on all of these 12 weeks and the AAC is the average of these values. The season starts on week 18, ends on week 28, and the season length is 10 weeks. The value of SPIn is the sum of pollen concentrations from week 18 to week 28.

## Supplementary Information

Supplementary Information.
