# Supplementary material for: Increased duration of pollen and mold exposure are linked to climate change
Source: Sci Rep. 2021 Jun 17;11:12816. doi: 10.1038/s41598-021-92178-z (PMC8211740; doi:10.1038/s41598-021-92178-z)
Supplement: Supplementary file 1 — Supplementary Information. [file 41598_2021_92178_MOESM1_ESM.docx]

**Increased Duration of Pollen and Mold Exposure are Linked to Climate Change**

Bibek Paudel^1^, Theodore Chu^2^, Meng Chen^1^, Vanitha Sampath^1^, Mary Prunicki^1^* and Kari C. Nadeau^1*^

## **Supplementary Appendix**

Timeseries decomposition

### For seasonal trend analysis of the time-series datasets, an additive decomposition method was applied, where the time series is modeled by a sum of three components: trend-cycle, season, and residuals, i.e., D(t) =T r(t) +S(t) +R(t). A frequency of 52 (corresponding to each week of data) was used in the decomposition and each decomposition was plotted in Supplementary Figure s5-s8.


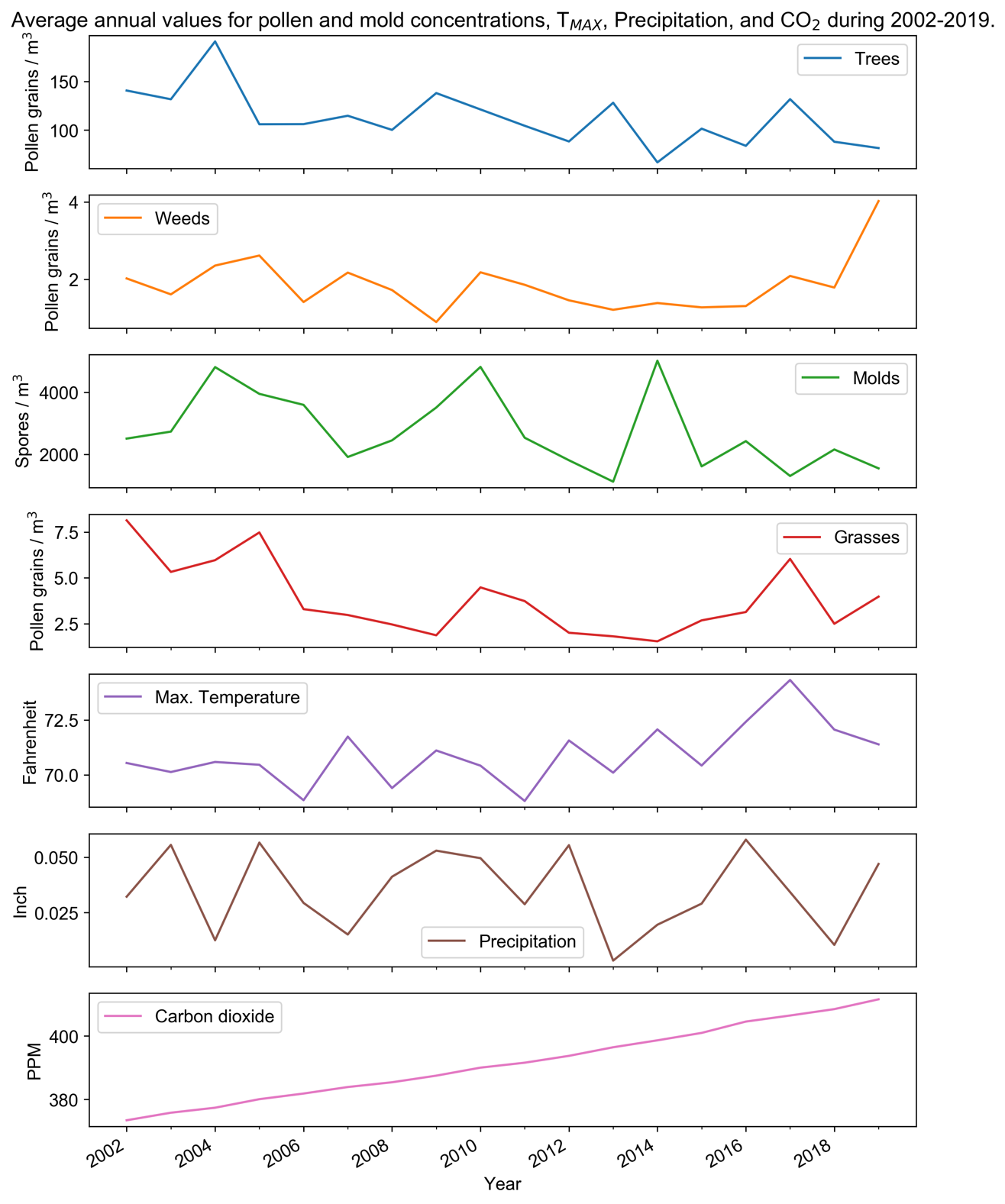


Figure s1. Average annual values for major allergens, T_MAX_, Precipitation, and CO_2_ values.


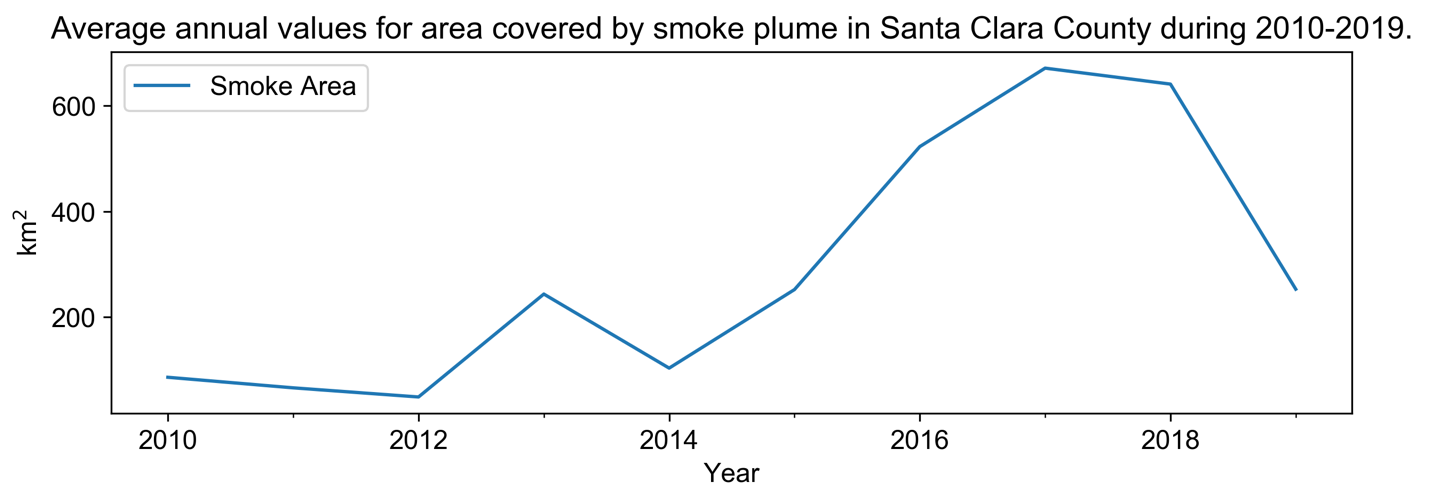


Figure s2. Average annual values for Smoke Area in Santa Clara County.


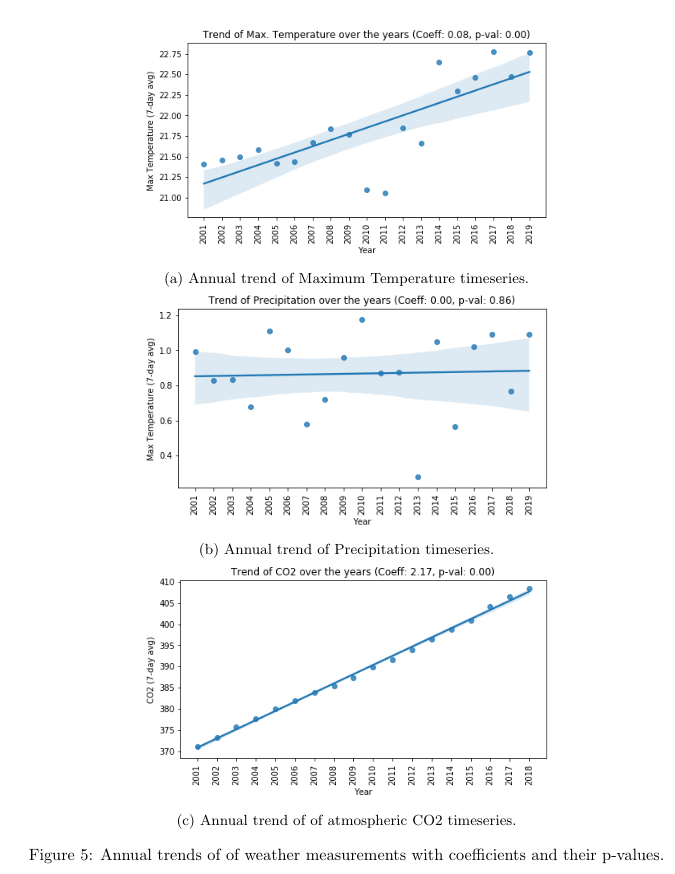


Figure s3. Annual trends of climate measurements with coefficients and their p-values.


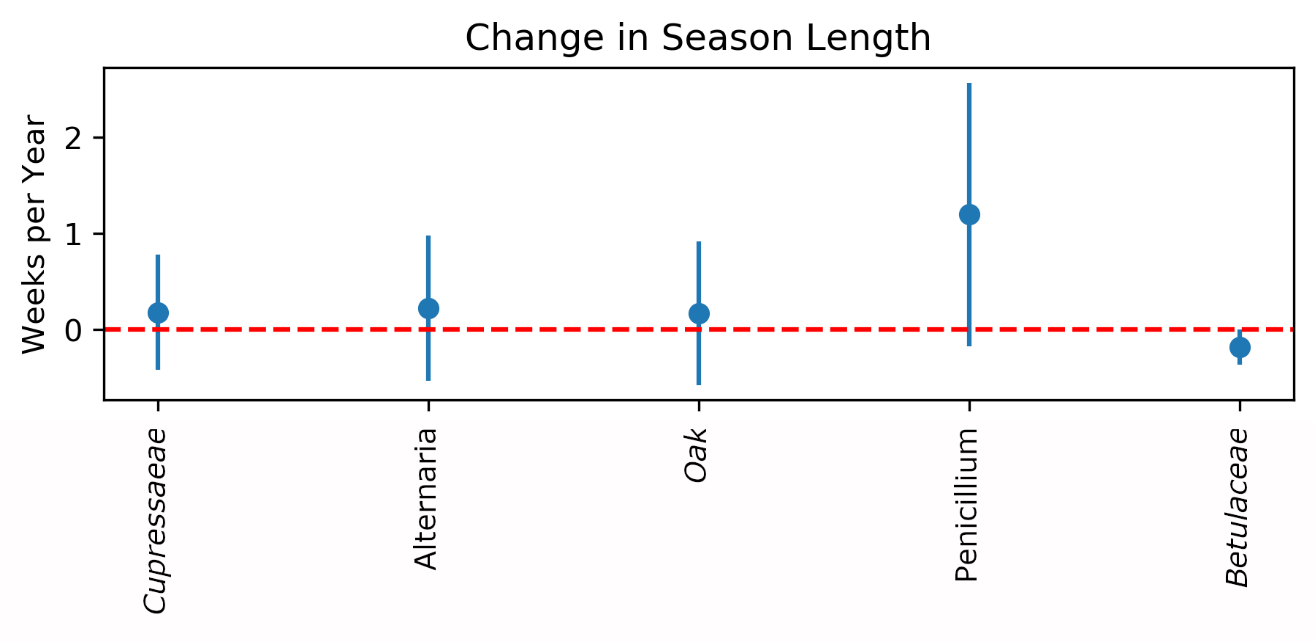


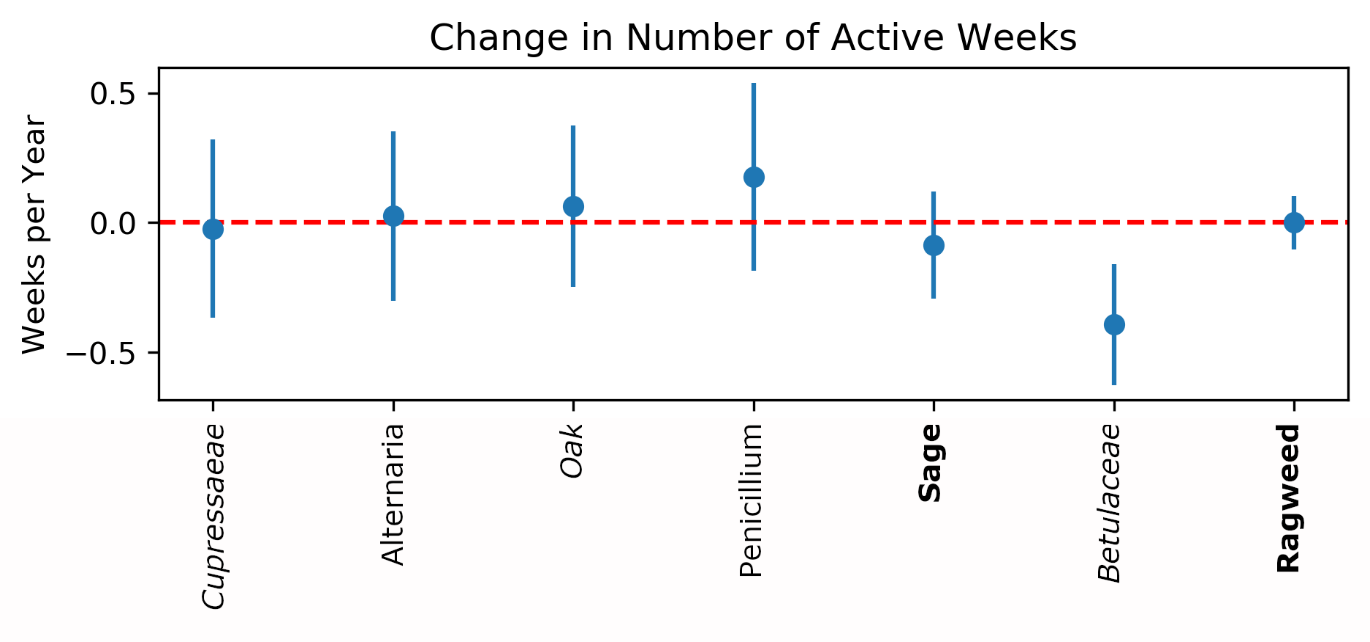


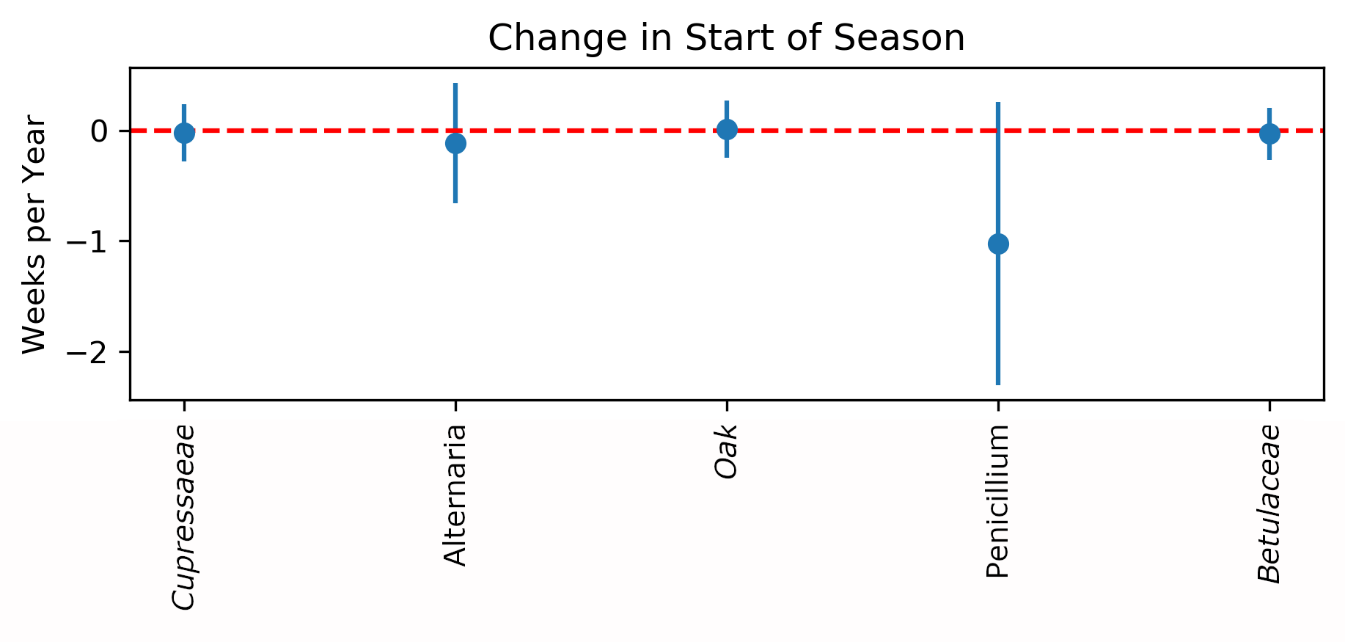


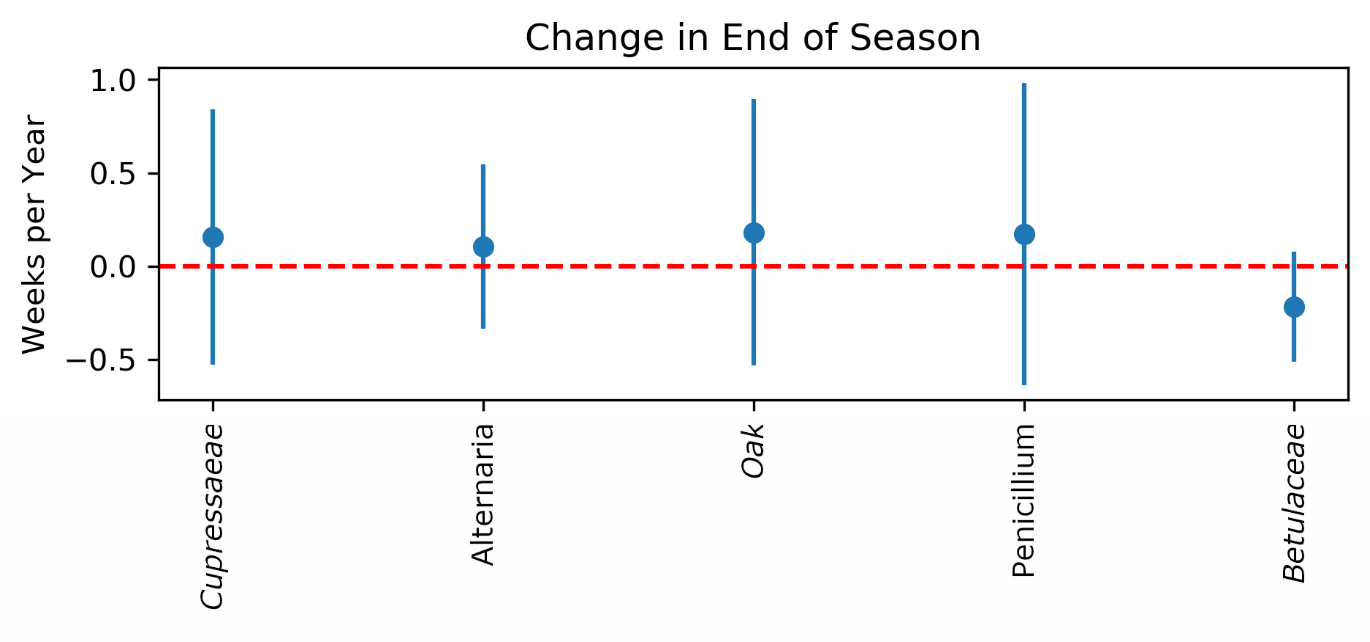


Figure s4. Coefficient estimates and 95% confidence intervals for change in season length, number of active weeks, start of season, and end of season for the selected species. Only those species for which season could be calculated for at least ten years are shown. Italics: *Trees*, Normal: Molds, Bold: **Weeds.**


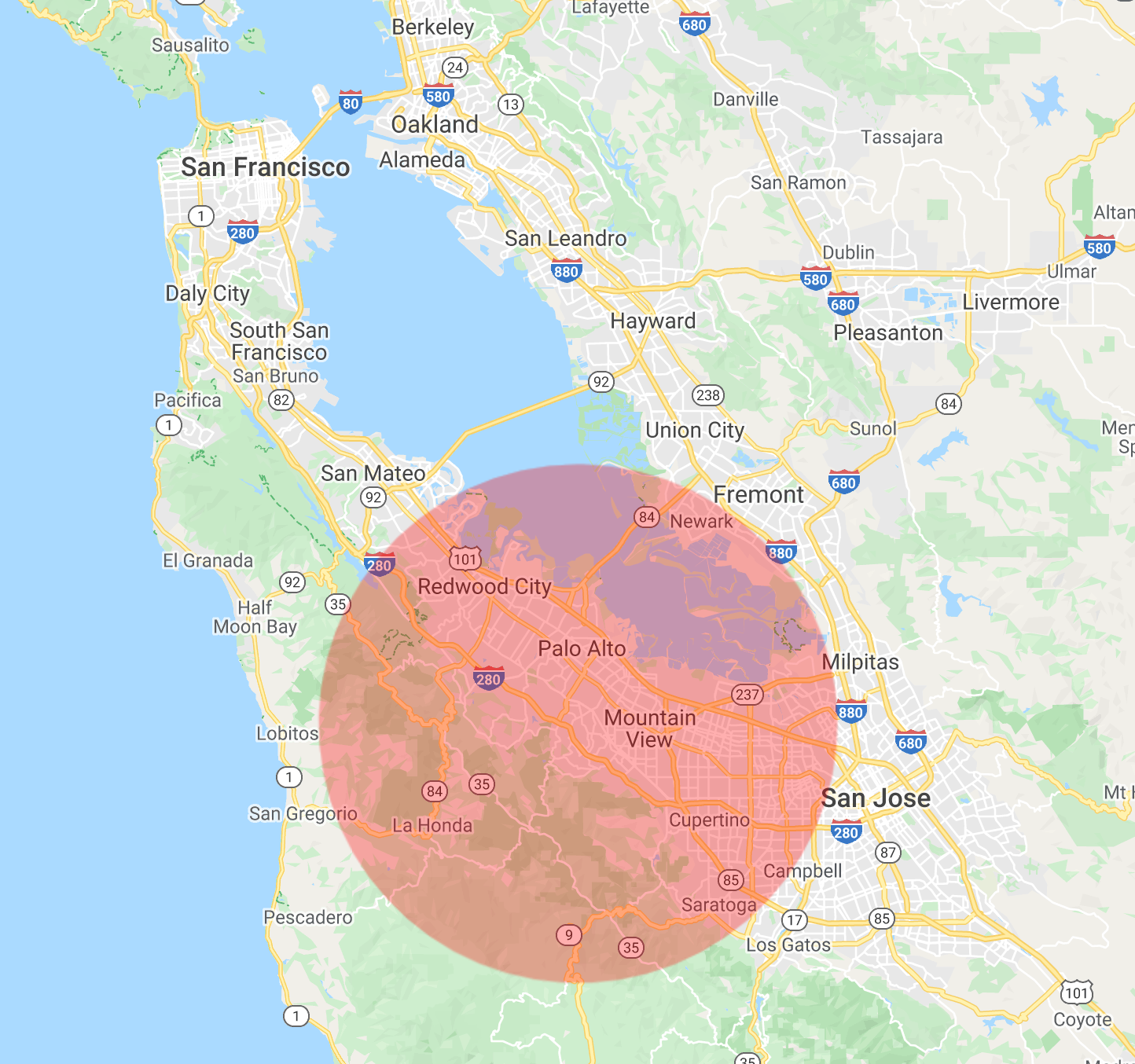


Figure s5. Spatial neighborhood (20 km radius) around the pollen data collection site. Map annotated using the Google Earth Engine Code Editor. This figure was generated using Google Earth Engine Code Editor https://code.earthengine.google.com/.


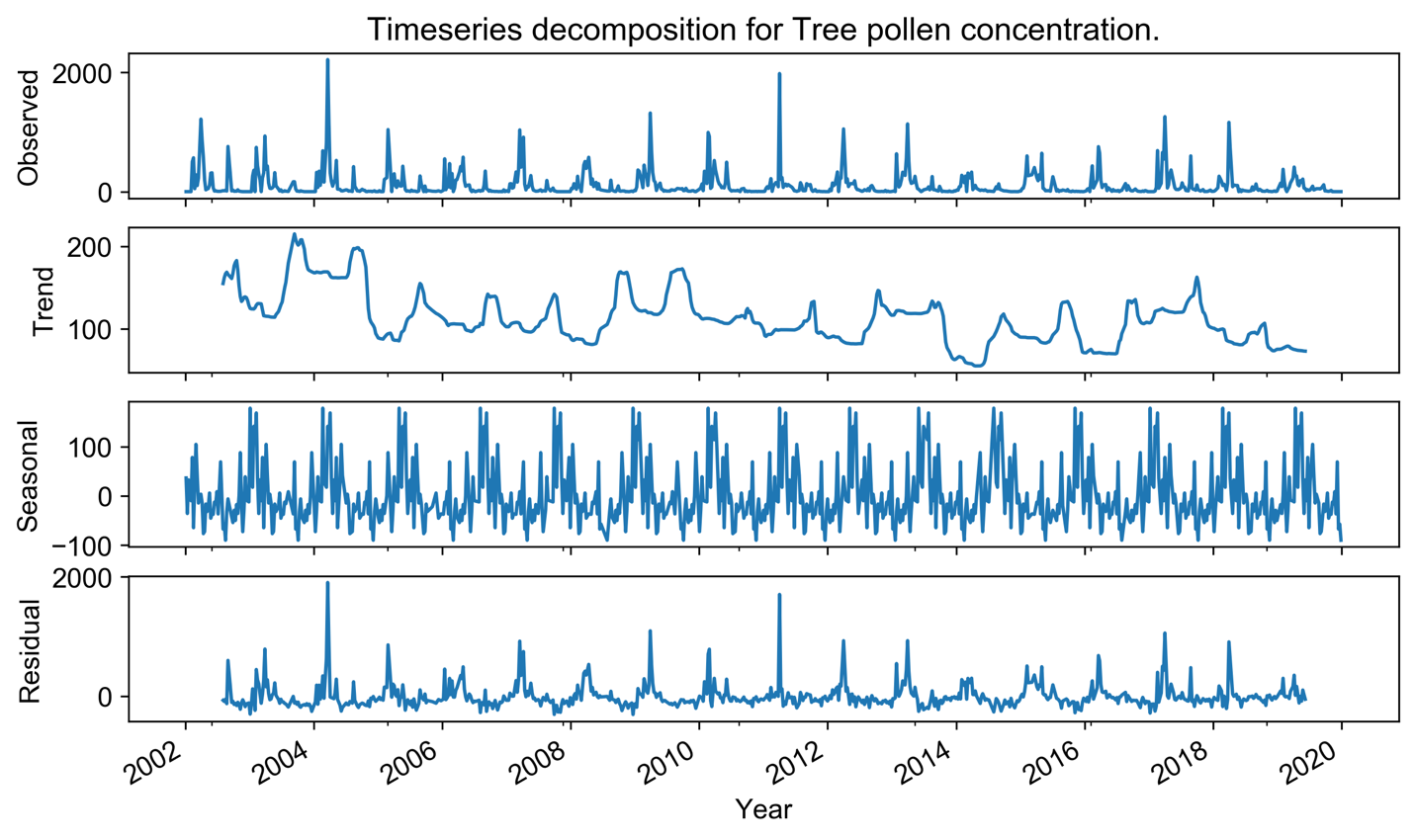


Figure s6. Seasonal decomposition of Tree pollen timeseries dataset (2002-2019).


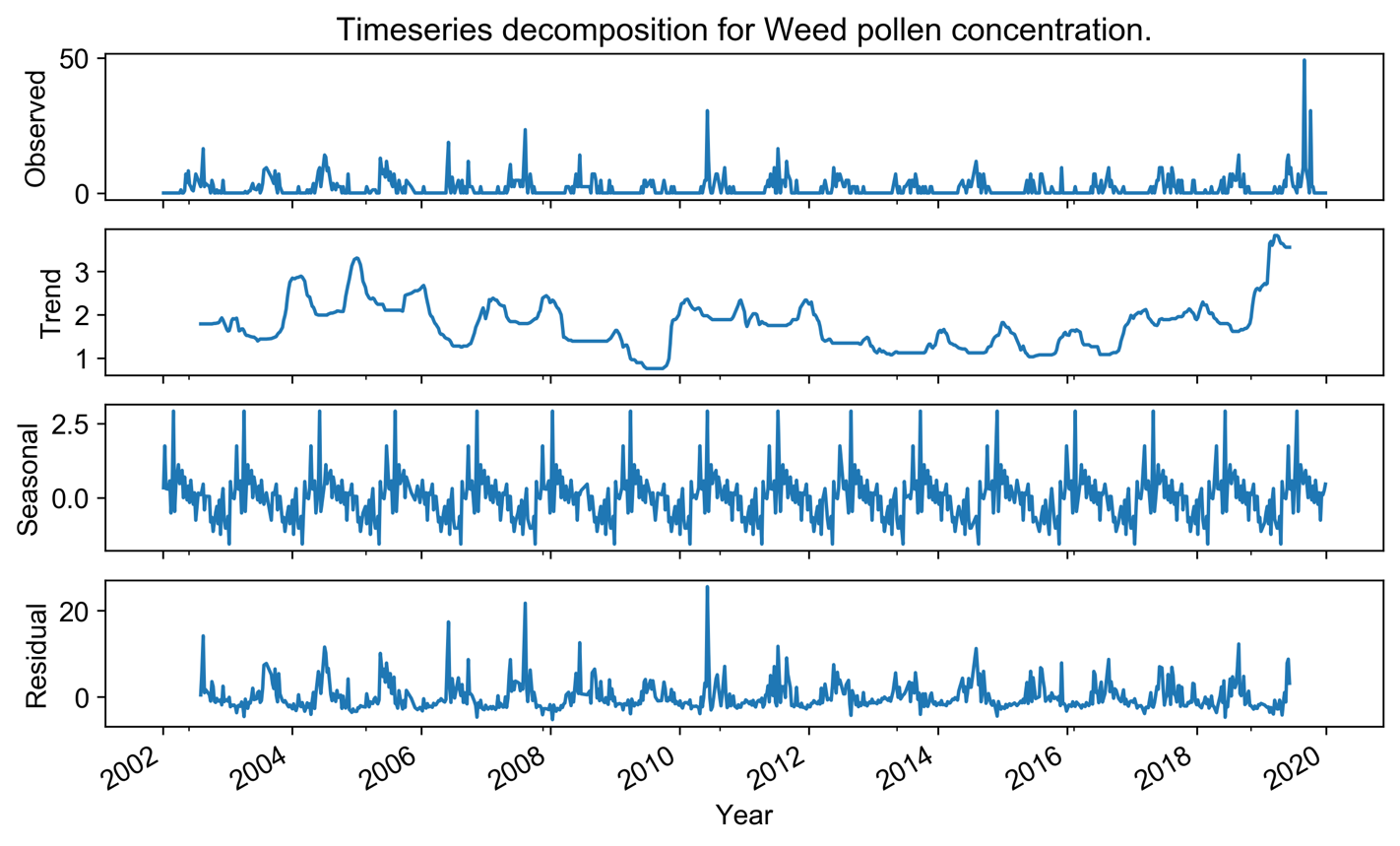


Figure s7. Seasonal decomposition of Weed pollen timeseries dataset (2002-2019).


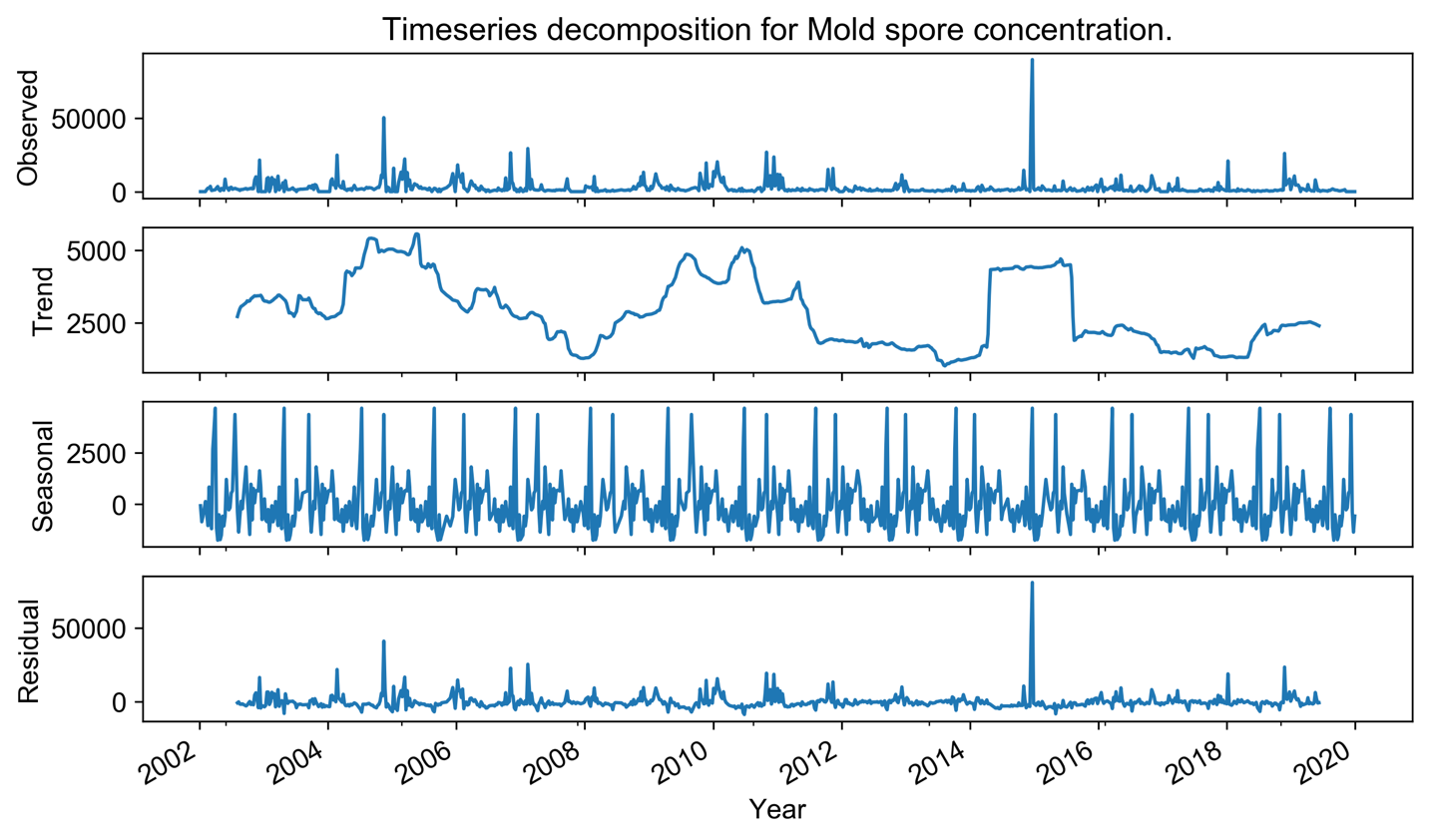


Figure s8. Seasonal decomposition of Mold spores timeseries dataset (2002-2019).


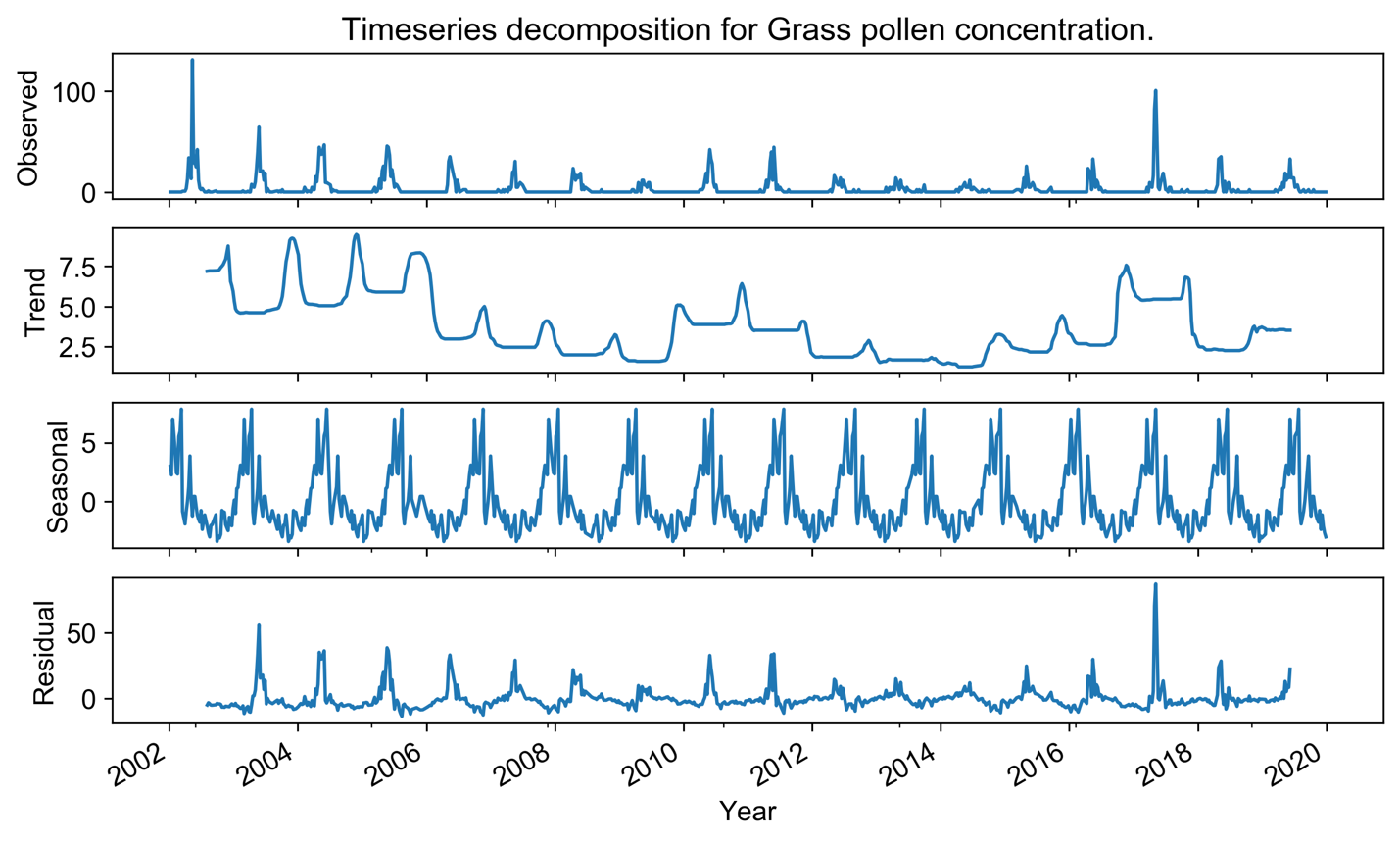


Figure s9. Seasonal decomposition of the Grass pollen timeseries dataset (2002-2019).


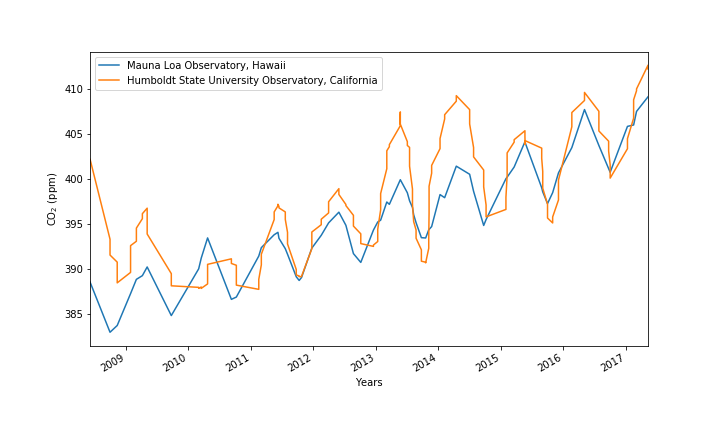


Figure s10. Comparison of the CO2 values (seven-day moving averages) at the Mauna Loa Observatory, Hawaii and Humboldt State University, California.


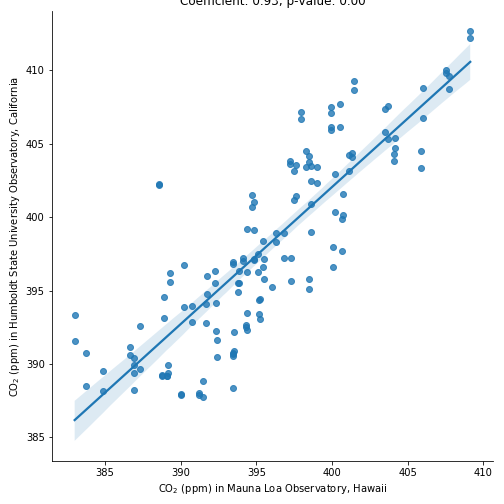


Figure s11. Linear relationship between CO2 values (seven-day moving averages) during 2008-2017 at the Mauna Loa Observatory, Hawaii and Humboldt State University, California (Correlation coefficient 0.93, p value 0.00).

Table s1. Temporal changes in the concentration and activity of major allergens (2002-2019), showing average change per year. A positive value for start of season or end of season indicates later start or end, and a negative value indicates earlier start or end. Active weeks are the weeks during which the concentrations were greater than zero for each type. Statistical significance (p value < 0.05) is denoted by an asterisk (*) and was ascertained using linear regression (see Methods section).

| **Type** | **Average rate of change of pollen/spore**  **concentrations per year** | **Average rate of change of pollen/spore season length, in weeks per year** | **Average rate of change of pollen/spore active weeks per year** | **Average rate of change of season start week**  **(Week**  **of the year)** | **Average rate of change of season end**  **(Week**  **of the year)** |
| --- | --- | --- | --- | --- | --- |
| **Trees** | -3.16* | 0.38* | 0.47 * | -0.08 | 0.29* |
| **Weeds** | 0.01 | -0.40 | -0.30 | 0.29 | -0.11 |
| **Molds** | -104.87 | 0.32 | 0.51 * | -0.08 | 0.23 |
| **Grasses** | -0.19 * | -0.08 | -0.21 | 0.10 | 0.01 |

Table s2. Temporal changes in the concentration and activity of selected species (2002-2019), showing average change per year. A positive value for start of season or end of season indicates later start or end, and a negative value indicates earlier start or end. Active weeks are the weeks during which the concentrations were greater than zero for each type. Statistical significance (p value < 0.05) is denoted by an asterisk (*) and was ascertained using linear regression (see Methods section).

|  | **Species** | **Average rate of change of pollen/spore**  **concentration per year** | **Average rate of change of pollen/spore season length, in weeks per year** | **Average rate of change of pollen/spore active weeks per year** | **Average rate of change of season start week**  **(Week**  **of the year)** | **Average rate of change of season end**  **(Week**  **of the year)** |
| --- | --- | --- | --- | --- | --- | --- |
| **Molds** | ***Alternaria*** | 0.19 | 0.20 | 0.003* | -0.01 | 0.19 |
|  | ***Penicillium/***  ***Aspergillus*** | 0.7 | 0.46 | 0.32 | -0.31 | 0.15 |
| **Trees** | ***Quercus spp* (oak)** | -0.39 | 0.30 | 0.02 | -0.13 | 0.17 |
|  | ***Cupressaeae spp* (incl. junipers/cedars)** | -0.58 | 0.21 | 0.12 | -0.02 | 0.19 |
|  | ***Betulaceae spp* (birch**  **and look alikes)** | -0.16* | -0.08 | -0.43* | -0.07 | -0.14 |
| **Weeds** | **Artemesia spp**  **(sage)** | 0.01 | 0.50 | -0.15 | -0.44 | 0.06 |
|  | ***Ambrosia spp/***  ***Franseria spp* (ragweed)** | 0.02 | 0.40 | -0.01 | -0.67 | -0.27 |

Table s3. Temporal changes in the concentration and activity of most commonly observed species (2002-2019), showing average change per year. A positive value for start of season or end of season indicates later start or end, and a negative value indicates earlier start or end. Active weeks are the weeks during which the concentrations were greater than zero for each type. Statistical significance (p value < 0.05) is denoted by an asterisk (*) and was ascertained using linear regression (see Methods section).

|  | **Species** | **Average rate of change of pollen/spore**  **concentration per year** | **Average rate of change of pollen/spore season length, in weeks per year** | **Average rate of change of pollen/spore active weeks per year** | **Average rate of change of season start week**  **(Week**  **of the year)** | **Average rate of change of season end**  **(Week**  **of the year)** |
| --- | --- | --- | --- | --- | --- | --- |
| **Molds** | **Ascospores** | -23.67 | 0.32 | 0.51 * | -0.08 | 0.23 |
|  | **Cladosporium** | -24.96 * | 0.36 | 0.48 * | -0.13 | 0.23 |
|  | **Basidiospores** | -55.54 * | 0.32 | 0.39 | -0.08 | 0.23 |
|  | **Unidentified Fungi** | -2.70 * | -0.27 | -0.32 | 0.46 | 0.19 |
|  | **Periconia** | 0.04 | -0.76 | 0.14 | 0.65 | -0.11 |
|  | **Rusts** | 0.12 | 0.54 | 0.08 | 0.08 | 0.62 |
|  | **Alternaria** | 0.19 | 0.20 | 0.00 * | -0.01 | 0.19 |
|  | **Algae** | -1.59 * | 0.15 | -1.14 * | -0.10 | 0.04 |
|  | **Smuts/Myxomycetes** | 0.12 | -0.31 | 0.07 | -0.33 | -0.64 |
|  | **Fusarium** | -0.28 | 0.79 | -0.19 | -0.11 | 0.68 |
|  | **Epicoccum** | 0.44 | -0.37 | 0.58 | 0.58 | 0.20 |
|  | **Oidium/Erysiphe** | -0.09 | -0.07 | -0.38 * | -0.32 | -0.39 |
|  | **Ganoderma** | 0.06 | 0.59 | 0.91 | -0.52 | 0.07 |
|  | **Coprinus** | 1.10 | 0.28 | 0.07 | -0.17 | 0.11 |
|  | **Torula** | -0.18 * | 0.20 | -0.51 | -0.52 | -0.29 |
| **Trees** | ***Pinus spp*** | -0.94 * | 0.79 | 0.19 | 0.06 | 0.85 |
|  | ***Cupressaeae spp* (incl. juniper/cedars)** | -0.58 | 0.21 | 0.12 | -0.02 | 0.19 |
|  | ***Ulmus spp* (elm)** | -0.36 | -0.50 | 0.06 | 0.58 | 0.08 |
|  | ***Quercus spp* (oak)** | -0.39 | 0.30 | 0.02 | -0.13 | 0.17 |
|  | ***Poaceae spp, Graminae spp* (Grasses)** | -0.09 | 0.08 | -0.05 | -0.06 | 0.01 |

Table s4. Top twenty most commonly observed species ranked by the total number of active weeks in each major category. Active weeks are the weeks during which the pollen/spore concentrations were greater than zero.

|  | **Species** | **Total number of active weeks (2002-2019)** |
| --- | --- | --- |
| **Molds** | Ascospores | 1,050 |
|  | Cladosporium | 1,047 |
|  | Basidiospores | 1,044 |
|  | Unidentified Fungi | 900 |
|  | Periconia | 746 |
|  | Ganoderma | 702 |
|  | Coprinus | 608 |
|  | Rusts | 585 |
|  | Alternaria | 533 |
|  | Algae | 456 |
|  | Smuts/Myxomycetes | 416 |
|  | Fusarium | 337 |
|  | Oidium/Erysiphe | 291 |
|  | Epicoccum | 289 |
|  | Torula | 287 |
| **Trees** | *Pinus* *spp* | 706 |
|  | *Cupressaeae* *spp* | 604 |
|  | *Ulnus spp* (elm) | 345 |
|  | *Quercus spp* (oak) | 305 |
| **Grasses** | *Poaceae spp, Graminae spp* | 366 |

**Table s5**

| **Category** | **Species (Trees, Weeds, Grasses)** |
| --- | --- |
| Trees | *Acer spp* (maple incl. box elder): *Acer Rubrum (*red maple) or *Acer Negundo* (box elder) |
|  | *Alnus spp* (alder): *Alnus rhombifolia* |
|  | *Betulaceae spp (birch and look alikes): Betula alba* |
|  | *Cupressaeae spp* (incl. juniper/cedars): *Juniperus occidentalis* |
|  | *Cyperaceae spp* (sedges) |
|  | *Fagus spp* (beech): *Fagus grandifolia* |
|  | *Quercus spp* (oak): *Quercus alba, Quercus rubra* |
|  | *Liquidambar spp* (sweet gum): *Liquidambar styraciflua* |
|  | *Carya spp* (hickory, pecan): *Carya ovata* (Hickory) |
|  | *Juglans spp* (walnut, butternut): *Juglans nigra* |
|  | *Morus spp* (mulberry): *Morus alba* |
|  | *Myrtaceae spp* (Eucalyptus family): *Eucalyptus globulus* |
|  | *Fraxinus* spp (ash): *Fraxinus velutina, Fraxinus americana* |
|  | *Olea spp* (olive): *Olea europaea* (Oleeae) |
|  | *Pinus spp* (pine and look alikes): *Pinus echinate* (yellow) |
|  | *Plantago spp* (English Plantain): *Plantago lanceolata* |
|  | *Platanus spp* (sycamore): *Platanus occidentalis* |
|  | *Populus spp* (poplar, cottonwood): *Populus alba* |
|  | *Salix spp (willow): Salix nigra* |
|  | *Ulmus spp* (elm): *Ulmus parvifolia* (Chinese elm), *Ulmus crassifolia* (Cedar elm), Ulmus pumila (Siberian), *Ulmus mericana* (American) |
|  | Douglas Fir: *Pseudotsuga menziesii* |
|  | Chinese Pistache: *Pistacia chinensis* |
| Weeds | *Ambrosia spp/Franseria spp* (ragweed): *Ambrosia psilostachya* (Western Ragweed) |
|  | *Artemesia spp* (sage): *Artemesia tridentata* |
|  | *Compositae spp* (aster family exc. Ragweed): *Xanthium strumarium* (cocklebur), *Salsola kali* (Russian thistle) |
|  | *Kochia scoparia* (firebush) |
|  | *Chenopodiaceae spp/Amaranthaceae spp:* *Chenopodium album* (lamb’s quarters), |
|  | *Fabaceae (legume=Acacia): Acacia dealbata, Acacia baileyana* |
|  | *Ligustrum (Privet): Oleeae Ligustrum vulgare* |
| Grasses | *Poaceae Graminae* (grasses): *Lolium perenne* (Ryegrass), *Phleum pretense* (Timothy), *Dactylis glomerata* (Orchard), *Cynodon dactylon* (Bermuda), *Poa Pratensis* (June, Kentucy blue), *Festuca pratensis* (Meadow fescue) |
|  | *Polyg Rumex* (sheep sorrel, dock): *Rumex crispus* (Yellow dock) |
|  | *Typha spp* (cattail) |
|  | *Urtica* (nettle) *Urtica dioica* |
| Molds | Ascospores |
|  | *Cladosporium spp* |
|  | Basidiospores |
|  | *Unidentified Fungi/Other Fungi* |
|  | *Periconia spp* |
|  | *Ganoderma spp* |
|  | *Coprinus spp* |
|  | Rusts |
|  | *Alternaria spp* |
|  | *Algae* |
|  | Smuts/Myxomycetes |
|  | *Fusarium spp* |
|  | *Oidium spp/Erysiphe spp* |
|  | *Epicoccum spp* |
|  | *Torula spp* |
|  | *Stemphylium spp* |
|  | *Penicillium spp/Aspergillus spp* |
|  | *Drechslera/Helminthosporium* |
|  | *Curvularia spp* |
|  | *Beltrania spp* |
|  | *Pithomyces spp* |
|  | *Polythrincium spp* |
|  | *Peronospora spp* |
|  | *Chaetomium spp* |
|  | *Erysiphe spp/oidium spp* |
|  | *Nigrospora spp* |
|  | *Smuts* |
|  | *Aspergillus* |
|  | *Paretaria (Pellitory)* |
|  | *Tilletia spp* |
|  | *Pestalotia spp* |
|  | *Calvatia spp* |
|  | *Eutypa lata* |
|  | Myxomycetes |
|  | *Cercospora spp* |
|  | *Agaricus spp* |
|  | *Beltrania querna* |
|  | *Bipolaris spp* |
|  | *Botrytis spp* |
|  | *Calvatia spp* |
